# 3. Stopping Hospital Infections with Environmental Services (SHINE): A Cluster-Randomized Trial of Intensive Monitoring Methods for Terminal Room Cleaning on Rates of Multidrug-Resistant Organisms (MDROs) in the Intensive Care Unit (ICU)

**DOI:** 10.1093/ofid/ofab466.003

**Published:** 2021-12-04

**Authors:** Matthew J Ziegler, Hilary Babcock, Hilary Babcock, Sharon F Welbel, David K Warren, William Trick, Sujan Reddy, Pam C Tolomeo, Jacqueline Omorogbe, Diana Garcia, Tracey Habrock-Bach, Onfofre T Donceras, Steven M Gaynes, Leigh Cressman, Jason P Burnham, David A Pegues, Ebbing Lautenbach, Jennifer Han

**Affiliations:** 1 University of Pennsylvania, Philadelphia, PA; 2 Washington University School of Medicine, St. Louis, MO; 3 Rush Presbyterian Hosptial, Skokie, IL; 4 Washington University, St. Louis, MO; 5 Cook County Health and Rush University Medical Center, Chicago, IL; 6 Centers for Disease Control and Prevention, Atlanta, GA; 7 Cook County Health, Chicago, Illinois; 8 John H. Stroger Hospital of Cook County, Chicago, IL; 9 Crothall Healthcare Inc., Philadelphia, PA; 10 University of Pennsylvania School of Medicine, Philadelphia, PA; 11 Washington University in St. Louis School of Medicine, St. Louis, MO; 12 Perelman School of Medicine at the University of Pennsylvania, Philadelphia, PA; 13 GlaxoSmithKline, Rockville, MD

## Abstract

**Background:**

MDROs frequently contaminate hospital environments. We performed a multicenter cluster-randomized, crossover trial of two methods for intensive monitoring of terminal cleaning effectiveness at reducing infection and colonization with MDROs within ICUs.

**Methods:**

Six medical and surgical ICUs at three medical centers received both intensive monitoring interventions sequentially, in a randomized order. The intervention included surveying a minimum of 10 surfaces each in 5 rooms weekly, after terminal cleaning, with adenosine triphosphate (ATP) monitoring or an ultraviolet fluorescent marker (UV/F). Results were delivered to environmental services (EVS) staff in real-time, with failing surfaces recleaned. The primary study outcome was the monthly rate of infection or colonization with MDROs, including methicillin-resistant *Staphylococcus aureus*, *Clostridioides difficile*, vancomycin-resistant Enterococcus, and multidrug-resistant gram-negative bacilli (MDR-GNB), assessed during a 12-month baseline comparison period and sequential 6-month intervention periods, separated by a 2-month washout. Outcomes during each intervention period were compared to the combined baseline period plus the alternative intervention period using mixed-effects Poisson regression, with study hospital as a random effect.

**Results:**

The primary outcome rate varied by hospital and ICU (Figure 1). The ATP method was associated with a relative reduction in the incidence rate of infection or colonization with MDROs (incidence rate ratio (IRR) 0.887, 95% confidence-interval (CI) 0.811–0.969, P=0.008) (Table 1), infection with MDROs (IRR 0.924, 95% CI 0.855–0.998, P=0.04), and infection or colonization limited to multidrug-resistant MDR-GNB (IRR 0.856, 95% CI 0.825–0.887, P< 0.001). The UV/F intervention was not associated with a statistically significant impact on these outcomes. Room turn-around time was increased by a median of one minute with the ATP intervention and 4.5 minutes with the UV/F intervention compared to baseline.

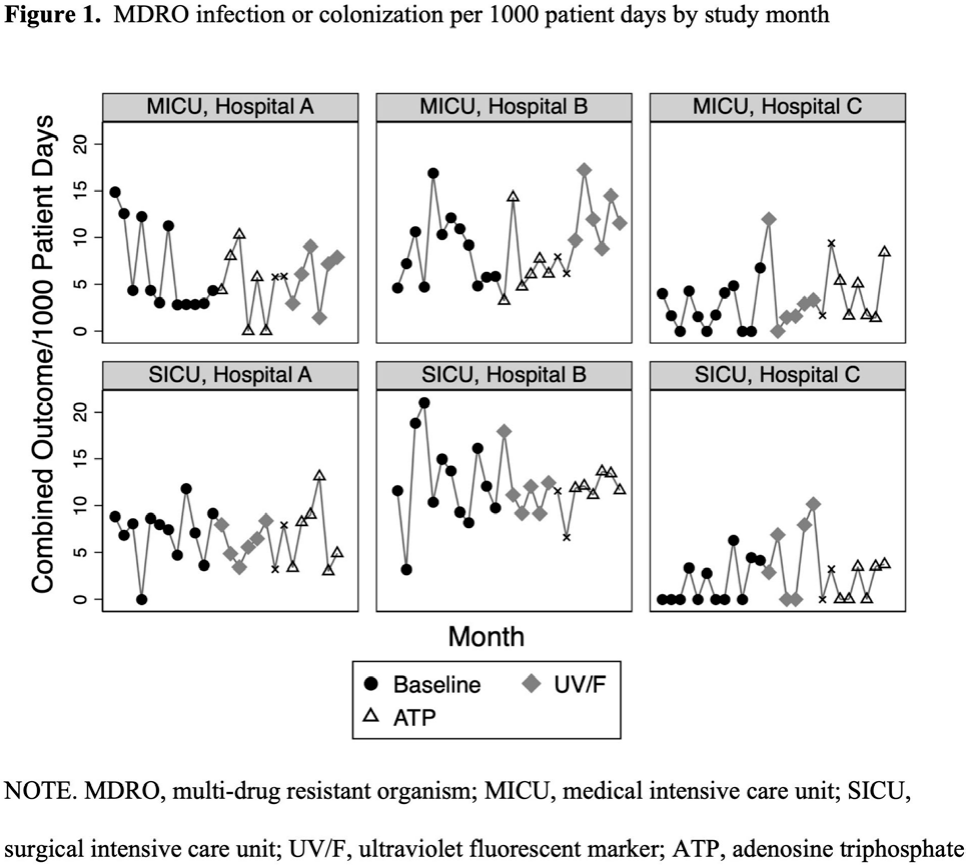

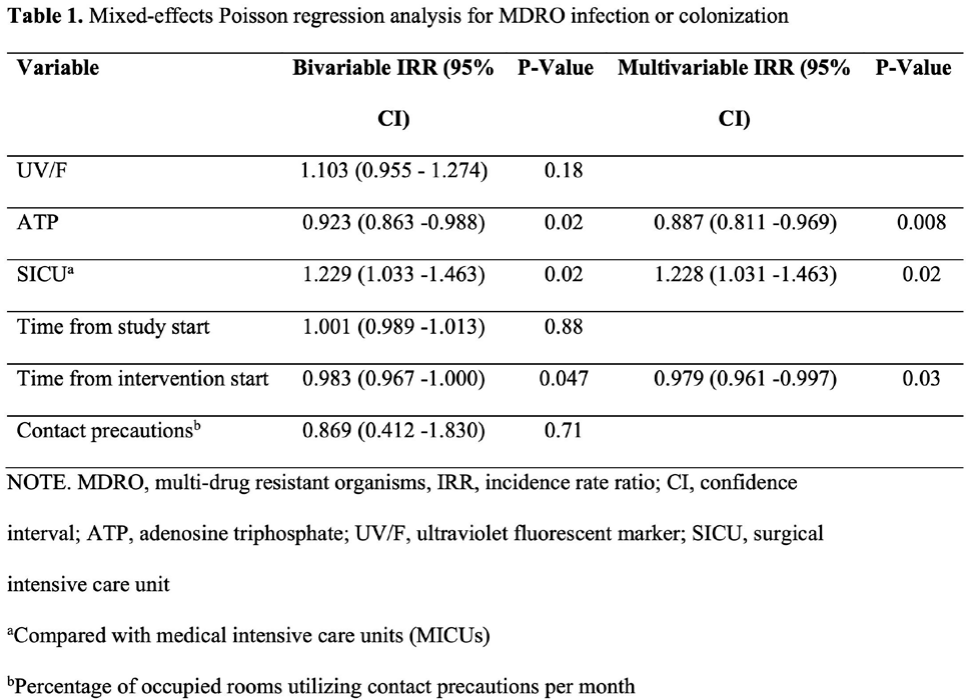

**Conclusion:**

Intensive monitoring of ICU terminal room cleaning with an ATP modality is associated with a relative reduction of infection and colonization with MDROs with a negligible impact on TAT.

**Disclosures:**

**Hilary Babcock, MD, MPH, FIDSA, FSHEA** (nothing to disclose), **David K. Warren, MD, MPH**, **Homburg & Partner** (consultant), **Ebbing Lautenbach, MD, MPH, MSCE** (nothing to disclose), **Jennifer Han, MD, MSCE**, **GlaxoSmithKline** (employee, shareholder).

